# The Impact of School Meal Programs on Educational Outcomes in African Schoolchildren: A Systematic Review

**DOI:** 10.3390/ijerph19063666

**Published:** 2022-03-19

**Authors:** Caitlin Wall, Terezie Tolar-Peterson, Nicole Reeder, Marina Roberts, Abby Reynolds, Gina Rico Mendez

**Affiliations:** 1 Department of Food Science, Nutrition and Health Promotion, Mississippi State University, Starkville, MS 39762, USA; cw2693@msstate.edu (C.W.); nr657@msstate.edu (N.R.); mr2447@msstate.edu (M.R.); amr503@msstate.edu (A.R.); 2 Social Science Research Center, Mississippi State University, Starkville, MS 39762, USA; gina.mendez@ssrc.msstate.edu

**Keywords:** school feeding program, education, education outcomes, attendance, enrollment, preschool, primary school, Africa

## Abstract

Malnutrition and hunger can lower a child’s ability to learn effectively. Many countries in Africa experience high rates of childhood undernutrition, and school feeding programs are a common tool used to address this challenge. A systematic review was conducted to evaluate the effect of school-provided meals on educational outcomes in preschool and primary school children. Specific outcomes of interest in this review included test scores, attendance, and enrollment rates. PubMed and Scopus were used for an electronic search of relevant studies. Studies included in this review were randomized and non-randomized controlled trials, prospective cohort studies, controlled before-after studies, and pre/post-test design studies published in the past 10 years in English in sub-Sahara Africa. Findings from the nine studies included in this review suggest a positive correlation between school feeding programs and educational outcomes. Although mealtime may reduce classroom time, the benefits of providing a meal outweigh the potential loss of learning time because hungry children may not learn as effectively. In conclusion, it is recommended that school meal programs be implemented and expanded. To improve general wellbeing and learning capabilities of children, school meals should be employed starting at a young age. More research on school feeding programs is needed concerning the preschool age group (2–5 years), as there is a limited amount of information in this area.

## 1. Introduction

More than 73 million children go to school hungry every day [1]. Children who do not have access to adequate nutrition may experience undernutrition associated with 45% of deaths of children under 5 years of age annually [2,3]. Additionally, adequate nutrition is an important part of a child’s development. Children under the age of 5 years old who are malnourished may have stunted cognitive development and learning capabilities into their adolescent and adult life [4]. As of 2019, 260 million children were not in school; these children will not develop the skills needed to succeed in life and the workforce [5]. Educational attainment is an important step to improving socioeconomic status and lowering poverty, making it an important area to address in sub-Saharan Africa, where a high percentage of children experience malnutrition, including wasting, stunting, micronutrient deficiencies, and overnutrition [6,7]. 

In 2000, the United Nations set Millennium Development Goals (MGDs) to be achieved by 2015 to combat poverty, hunger, and low educational achievement [8]. The MDGs have been superseded by the Sustainable Development Goals (SDGs) to be achieved by 2030 [8]. Food for education programs are being used to reach MDGs and SDGs [9]. School feeding programs may also provide vital micronutrients that may benefit cognitive abilities and educational outcomes [9,10]. The two main types of food for education programs are school feeding programs (SFP) and take-home ration (THR) programs. School feeding programs typically offer a breakfast and/or lunch meal during the school day, while THR programs provide a certain number of commodities to the student’s household per period conditional on attendance [11]. School feeding programs have the potential to alleviate short term hunger to increase concentration and learning capabilities [9]. In younger children, it is up to the parents to decide whether to enroll a child in school; parents may base the decision on the perceived value of education or the direct cost of schooling [12]. School feeding programs offer benefits by providing children with food to relieve the burden of providing meals at home, which in turn may motivate parents to enroll their children; however, parents of children in these programs may redirect food at home to children that are not getting the meal at school [11]. While there is some debate on the effectiveness of school meal programs on learning outcomes, a study in Uganda found a significant correlation between academic performance and meal consumption [13]. A program implemented in preschools in Mozambique improved cognitive function and increased primary school enrollment [14]. Breakfast consumption has been shown to improve cognitive function and educational outcomes even in populations that are not severely malnourished [15]. While it has been established that having a sufficient diet can improve academic outcomes and cognition, it is still unclear whether the nutrients provided by school feeding programs are enough to influence learning capabilities [16].

The primary objectives of this research were to determine if SFPs have a substantial effect on educational outcomes and whether the potential effect is positive. With new programs being implemented at an increasing pace, it is crucial to determine what methods lead to the best outcomes. This review serves as an identification and analysis of current literature to prepare for future school meal program implementation.

## 2. Materials and Methods

### 2.1. Background

This review was conducted to synthesize and analyze the research relating to the impact of school meal intervention programs on educational outcomes among children in preschool and primary school in sub-Saharan Africa. The search strategy for this review followed the Preferred Reporting Items for Systematic Reviews and Meta-Analyses (PRISMA) guidelines [17]. The PRISMA checklist can be found in the supplementary materials. There was no protocol prepared for this review. Databases accessed for this review were PubMed and Scopus and were searched in May 2021. 

### 2.2. Eligibility Criteria

Articles published in English between 2011–2021 were eligible for review to keep the data more relevant. Only studies from the past ten years were considered in this review seeking relevance to current SFP efforts. Study designs considered for inclusion were randomized and non-randomized controlled trials, prospective cohort studies, and before-after studies. This review had a target population of African preschool (age 3–5 years) and primary school (age 6–12 years) students, as less is known about the effect of SFPs on children in this age range. Some studies included in this review included children older than 12 years of age because they were still attending primary school at the time of the study. To be included in this review, articles must include data and discussion of educational outcomes; articles that primarily focused on nutrition and anthropometric outcomes were not considered relevant. Table 1 gives an overview of the criteria used when deciding to include a source. 

### 2.3. Search Strategies

Search terms were tested prior to the final collection to determine relevancy. Terms included in the search for both PubMed and Scopus included “school meal”, “attendance”, and “education”. The complete list of search terms can be found in Appendix A. All results were imported into citation management software to eliminate duplicates. The remaining articles were advanced to the first screening using Rayyan [18]. Following the pre-determined inclusion criteria, articles were first assessed by one researcher based solely on title and abstract. A second round of screening was conducted based on the full-text articles. During the second round of screening, four independent researchers assessed each article for eligibility and relevance. Conflicts were discussed until an agreement was reached. Data extraction was completed by one researcher and compiled into a summary table. Specific outcomes for which data were collected included attendance rates, enrollment rates, and academic performance. Studies that assessed cognitive development were grouped with studies that reported changes in academic performance because both measures were obtained by assessing test scores. Some studies additionally measured outcomes related to anthropometric measurements, and while that data was available, it was not the focus of this review. 

### 2.4. Certainty of Evidence and Risk of Bias

The risk of bias and quality of evidence was assessed using the Academy of Nutrition and Dietetics Quality Criteria Checklist (QCC) tool [19]. The QCC for risk of bias consists of 10 questions that determine the validity of the source, with the first four questions being the most critical. Based on the answers to the questions, each article is given a positive, negative, or neutral rating. The QCC for quality of evidence consists of five questions. Each article is assessed for each question and given a grade of I-V. Three researchers evaluated each article individually and discussed any differences until an agreement was reached. For this review, 8 out of 9 articles were rated positively for risk of bias, with the final source being negative. Concerning the quality of evidence, 8 of the 9 articles were rated high-quality, and one article was rated as moderate quality. A complete evaluation is included in Appendix A.

## 3. Results

### 3.1. Study Selection and Characteristics of Included Studies

**Figure 1:**
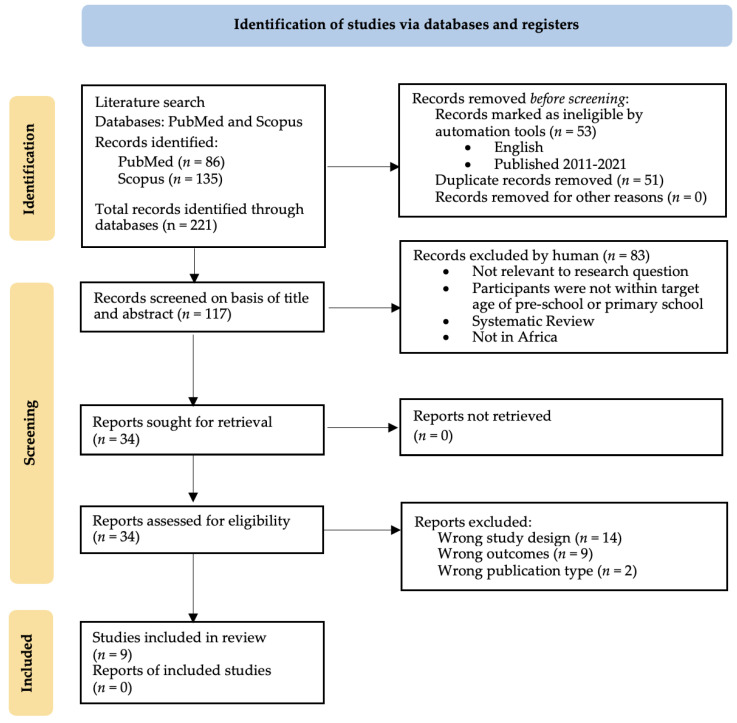
PRISMA screening process for selection of articles for review [17].

From the two databases, 221 articles were identified, and 170 remained after excluding duplicates. Of the 170 remaining unique articles, 52 were excluded by automated tools because the primary language was other than English, or because the publication date lay outside the past 10 years. The 117 articles moved to the first screening process were assessed based on title and abstract, and 83 were excluded as they did not meet the inclusion criteria. Of the 34 articles assessed based on full-text evaluation, 9 were included in this review. Three independent researchers decided on the included articles using Rayyan [18]. If there was a conflict, the articles were discussed until a decision was reached. A conceptual map for this process can be found in Figure 1. Table 2 summarizes the included articles with information on study design, study population, study duration, intervention details, outcomes measured, and the main findings of each study. Of the studies included, 3 were randomized controlled trials (RCT), 2 were cluster randomized controlled trials (CRCT), 1 was a prospective cohort study, and 3 were pre-posttest design. All studies were conducted in sub-Saharan African countries, including Uganda, Senegal, Ethiopia, Kenya, South Africa, Burkina Faso, and Malawi. The intervention in all studies was some form of food supplementation, whether it be a meal during class or take-home rations. 

### 3.2. Attendance

Five of the nine studies included outcomes related to attendance [11,20,22,24,26]. Of these, two studies compared SFP and THR interventions [11,20], one compared a school with an SFP to a school without an SFP [22], one implemented a THR only [24], and one added a mid-morning snack [26]. All five studies reported benefits to attendance rates for students who received food. 

The first study that compared SFP and THR programs, a CRCT conducted among displaced people camps in Northern Uganda, compared attendance rates between students that received an in-school meal versus students that received a monthly THR [20]. Attendance was taken by research assistants who made unannounced visits to the schools. Older students aged 10–17 had improved attendance rates for the morning sessions (8% increase for the SFP group and 12% increase for the THR group). Afternoon attendance improved for children aged 6–17 by 14.6% and 14.1% in the SFP and THR groups, respectively [20]. The second study that included both an SFP and a THR intervention group was conducted in Northern Burkina Faso, a region with one of the world’s lowest rates of primary school participation [11]. This randomized trial reported improved attendance in the SFP group by an average of 0.7 school days over the previous month. The effect of attendance by THR provision was an increase of 0.9 days over the previous month. However, when the data was restricted to children who first enrolled in school at the beginning of the study, there were no significant effects on attendance for boys, and girls’ attendance decreased by one day [11]. In this instance, the decrease in attendance is believed to result from program motivated enrollment [11], as students who would not have enrolled without the promise of the meal may be less motivated to attend school. 

An RCT that examined the effect of an SFP versus no SFP on the attendance of students in Senegal provides further support for the effect of school meals on attendance [22]. It was found that students who did not receive daily school meals were two times more likely to miss class [22]. While SFP students missed an average of 4 days during the school year, non-SFP students missed an average of 9.3 days, suggesting that daily school meals do increase attendance compared to not receiving any meals at all [22]. A study in Burkina Faso looking at the effect of a THR program only on attendance, attendance appeared to improve for both girls and boys when girls were given a THR [24]. Implementing food rations in rural primary schools in Northern Burkina Faso, where girls’ enrollment rates were less than 40%, resulted in a 6% increase in girls’ attendance and an 8.4% increase in boys’ attendance rates [24]. Finally, a study in Kenya analyzed the effect of three different morning snack compositions on attendance: a Meat githeri, a Milk githeri, and an isocaloric Plain-githeri [26]. Attendance in this study decreased across all groups, perhaps related to a severe drought that occurred during the study period; however, children in the intervention groups still had slightly higher attendance rates than children not receiving any food at school, attending an average of 2% more school days [26].

### 3.3. Enrollment

Four of the nine studies included enrollment as an outcome [11,20,21,24]. Two of these compared SFP and THR interventions [11,20], one looked only at the effects of an SFP [21], and one looked only at the effects of a THR program [24]. In the randomized trial conducted in Burkina Faso, which compared the effects of an SFP and a THR on educational outcomes, enrollment increased by 4% for students in the SFP group and 4.8% for children in the THR group [11]. In THR schools, even boys in the program with no sisters to bring home rations saw an increase in enrollment [11]. Conversely, in the CRCT conducted in Uganda, which compared the effects of an SFP and a THR on educational outcomes among children in displaced people camps, there were no significant impacts of either program on enrollment [20]. However, in a sub-analysis restricted to only children who were not enrolled in school at baseline, the SFP resulted in a 9% increase in the probability of a child enrolling within the next two years [20]. On the other hand, the THR program did not have any significant effect on the enrollment of children who were not enrolled in school at baseline [20]. Furthermore, an SFP intervention implemented in schools in four rural regions of Senegal found no significant effect of equipping schools with canteens on enrollment for the targeted population, which was second and fourth graders [21]. While there was a small increase in enrollment, it was not enough to be considered a valid outcome [21]. It should be noted, however, that the school meal program in this rural Senegal study contained a high degree of heterogeneity, with some schools only providing meals two days a week and other schools providing meals up to five days a week [21]. Finally, in the fourth study, enrollment rates increased 3.2% for girls in Burkina Faso when they were provided a THR contingent on consistent attendance [24]. Overall, the effect of school feeding programs on enrollment rates was inconsistent, with neither school meals nor take-home ration programs appearing to have a significant advantage over the other at improving enrollment rates.

### 3.4. Academic Performance

Six of the nine studies included data about academic performance [11,16,21,22,23,25]. All six studies included some form of food at school, whether breakfast [16], a mid-morning snack [23], or lunch [11,21,22,25], and one study included a THR group in addition to the SFP [11]. In the RCT conducted in Burkina Faso that compared the effects of SFP and THR programs, students in the SFP group increased the proportion of correctly answered math questions by 9.6%, and students in the THR program produced an increase of 8.4% [11]. Girls’ scores improved by 11.3% and 9.4% in the SFP and THR program, respectively. Boys saw a significant increase only in SFP schools, with an improvement of 7.9%. Participants in this study were also administered Raven’s Coloured Progressive Matrices test, an assessment of abstract reasoning, and the Wechsler Intelligence Scale for Children, which assesses short-term memory. There was no significant difference between the treatment and control groups for cognitive development [11]. Looking at the three other studies that provided lunch at school, two of them also demonstrate a positive effect of SFPs on test scores [21,22], and one demonstrates a positive effect of SFPs on a cognitive function test [25]. In Senegal, aggregate, French, and Math scores significantly improved in the children served lunch two to five times a week. The differences in test scores between the SFP group and the control group were 5.5, 4.9, and 6.1 points in each subject area, respectively [21]. In Ethiopia, children in SFP participating schools were compared to children in non-SFP participating schools based on an aggregate academic score of 10 subjects [22]. Children in the SFP schools scored an average of 2.3% higher for their aggregate academic score than students without a meal program [22]. A benefit to cognitive function resulting from an SFP was observed in a study conducted in Malawi, which administered the Cambridge Neuropsychological Test Automated Battery (CANTAB) to assess memory, reversal learning, and attention [25]. Subsets of the CANTAB test used include paired associate learning (PAL), the intra-extra dimensional shift (IED), and rapid visual information processing (RVP). On the CANTAB test, the SFP cohort experienced a greater decrease in the intra-extra dimensional shift pre-extra dimensional errors, which assumes that the meal intervention can improve reversal learning [25]. 

Finally, two studies examined the effects of lunch alternatives: a school breakfast program and a morning snack program. Results from the school breakfast program were inconclusive, as scores improved for children in grade R through grade 3 with varying levels of improvement, but scores for children in grades 4–9 mostly declined [16]. It is suspected that a recent change in curriculum could have attributed to the decline in grades [15]. Grade 5 was the only upper-level class that saw a performance improvement. Despite this, many of the teachers and school staff who were interviewed believed that the program improved participation and concentration [16]. In the morning snack study, children were divided into three treatment groups to determine if animal-source protein impacted learning outcomes [23]. Students who received githeri with meat and githeri with milk had significant test score improvements compared to children in the Plain-githeri and control groups. The total point difference between term 1 and term 5 test scores across all subjects between the Meat-githeri group and the control group was 57.5 points, and the difference between the Milk-githeri group and control group across all subjects was 39.4 points. There was no significant difference in test scores between the plain githeri and control groups, suggesting that including animal-sourced foods in school feeding programs may provide additional benefits [23]. 

## 4. Discussion

The purpose of this systematic review was to analyze the impact of school meal programs on educational outcomes among schoolchildren residing in sub-Saharan Africa. Understanding how school meal interventions can improve learning outcomes is vital to improving the overall quality of life, as educated children will likely become more productive members of society [5]. The general trend among current literature finds that food for education programs improve education in at least one facet. Both in-school meal programs and take-home ration programs have found success. Whether it be attendance, enrollment, or test scores, 8 of the 9 studies in this review found that SFPs improve learner outcomes. While a South African study found education outcomes inconclusive, there were improvements in anthropometric measurements with a 10% increase in the number of children within the healthy BMI range for their age [16]. No study determined that the SFP harmed learner performance. There were some concerns that having a meal served at school would take away from education time [7,20]. However, the improvements in learning outcomes found in the studies included in this review suggest a net positive effect of school meals on educational outcomes. Although it does take time to serve and eat the food, the students are more productive during class time [16]. Concerning areas or curriculums that may find it difficult to accommodate a daily meal, a THR program should be no less effective [20]. If students are no longer distracted by hunger, the time spent in the classroom would be more effective. Children that consume a meal before learning have better short-term memory function as the brain activates differently based on nutrient supply [13]. While most programs provide a meal at lunch, meals served at breakfast are also shown to have a positive impact [16].

Based on the findings in this review, attendance is an acceptable indicator of academic improvement. If children are in classes more often, it can be assumed that they will learn more. Attendance is also a relatively easy indicator to measure, as it can be taken fairly quickly and frequently. There do, however, remain some challenges to strictly using attendance as an indicator of academic improvement. First, using attendance as an indicator of academic improvement relies on the assumption that students are learning more just by being present. It is possible that a student could be counted in the roll for the day but then leave after a meal is served. This would mean they were not present for all academic instruction, thus not acquiring the knowledge needed to improve their learning outputs. This problem might be solved by requiring an afternoon roll call, as a study from Uganda obtained more accurate attendance data by collecting morning and afternoon attendance rates [20]. Second, teachers and parents may overstate attendance rates because it would reflect poorly on them if children did not show up for class [20]. Finally, it must be taken into consideration that students who would not normally attend class may enroll in the school for the meal incentive after the implementation of an SFP, and this could lead to the attendance percentage being lower than expected [11]. Enrollment is not the best indicator for improved academic outcomes, as it does not necessarily indicate increased attendance. Having more children enrolled in classes does not help if they are not attending classes. It is also possible that schools may inflate their enrollment numbers to increase their funding, or that there may be students that attend class without being officially enrolled in the school [23]. The most concrete indicators of improved learning outcomes are measures of academic achievement such as cognitive or subject test scores. Comparing test scores can show direct improvements in learner performance. The increased intake of vital micronutrients directly impacts test scores and comparing test scores can show direct improvements in learner performance [23]. A drawback to assessing students with academic tests is time and money constraints. There may also be outside factors that can affect the validity of test scores. 

A limitation of this review is the lack of studies available in the preschool age group. This review was focused on the age groups 2–5 (preschool) and 6–12 years of age (primary school), but the majority of the studies were in the primary school age group. Many children were also older than typical primary school age due to grade repetition. There was a significant lack of information regarding the effect of school meal programs on educational outcomes for the preschool age group. This might be due to the lower number of children enrolled in preschools than primary schools, as primary education is often required in many countries. This leaves a significant gap in the literature between the first 1000 days of life and children above five years of age. Although the vast majority of research on malnutrition concerns children under three years of age, it is just as important to provide older children with essential nutrients as they are still developing. Undernourished children in the preschool age group who receive the micronutrient supplementation show improvements in cognitive development [10]. A second window for growth and cognitive development opens from age 5 to 19. During puberty, growth and height velocity are at their highest, so there are still benefits to providing meals to older children [2,27]. A strength of this review is the rigorous process of article selection and the high-quality nature of the studies assessed.

## 5. Conclusions

With the implementation of school feeding programs steadily increasing, it is imperative to continue to assess their impact on students. These programs are an integral part of the effort to reduce child hunger and malnutrition in low-income countries. The consensus of the results presented in this review suggests that school meal interventions improve educational outcomes in at least one aspect; attendance, enrollment, or learning capabilities. Providing children with a meal during the school day may encourage participation and increase concentration by reducing hunger as a distraction. With the development of more school feeding programs, it is also imperative to establish program goals and objectives at the start of implementation. Measuring outcomes against defined objectives would be more effective in measuring changes. The potential drawbacks of these programs, such as loss of teaching time, do not outweigh the improvements in educational outcomes achieved with the school meals. If loss of teaching time remains a concern, it may be easier to provide a THR program to avoid disruption during the school day. Even if the meals are not enough to improve the long-term nutritional status of the children, the short-term benefits can improve their education. It is recommended that current school meal intervention programs be expanded and new programs be implemented. It would be beneficial to start meal programs in preschool, as this may help with cognitive development and may encourage more parents to enroll their children in preschools. 

## Figures and Tables

**Table 1:** Criteria for article inclusion and exclusion.

*Criteria*	*Inclusion Criteria*	*Exclusion Criteria*
**Language**	English	Non-English
**Study Type**	Randomized and cluster randomized controlled trials, prospective cohort studies, controlled before-after studies, pre-posttest design	Systematic reviews, reviews, meta-analyses, protocols, methodologies, cross-sectional studies
**Date**	Published in the past 10 years	Published before 2011
**Population**	Children in African countries enrolled in preschool or primary school	Children in non-African countries; infants and children in grades above primary school
**Relevance**	Must include outcomes or data about educational outcomes, including attendance, reading comprehension, memory, enrollment, or literacy	Nutritional outcomes only Anthropometric data only

**Table 2:** Summary of included articles discussing the impact of school meal interventions on educational outcomes among preschool and primary school age children in Africa.

Reference	Study Design	Participants and Study Duration	Intervention	Outcomes Measured	Main Findings
Alderman et al. [20] (2012; Uganda)	CRCT	*n* = 31 internally displaced people camps in the Pader and Lira districts of Northern Uganda. Households with children 6–17 y were surveyed. Study duration = 18 months (initiated October 2005)	School feeding program (SFP): mid-morning snack of fortified porridge and a hot lunch of beans and maize or rice (1049 kcal/day) Take-home ration (THR): Households received THR for each primary school child enrolled and attending 85% of school days.	Enrollment, attendance, age at entry, grade repetition, and progression to secondary school	SFP resulted in 9% increase in probability that 6–13-year-old children not enrolled in school at baseline would enrol within two years. SFP & THR improved morning attendance for children ages 10+ (8–12% increase), and both improved afternoon attendance for children 6–17 y by ~14%.
Azomahou et al. [21] (2019; Senegal)	RCT	*n* = 120 primary schools in four rural regions of Senegal (Fatick, Diourbel, Kolda, and Sedhiou). Children in the second and fourth grades were sampled. Study duration = 16 months (initiated in 2009)	Canteen: a hot lunch consisting of maize, lentils, and fortified oil provided through the school canteen (699 kcal/day). Canteen + Deworming Deworming only	Test scores (French, math, and aggregate). Internal efficiency (enrollment, promotion, repetition, and dropout)	Canteens improved aggregate test scores by 6.37 percentage points. Aggregate and math scores improved more for girls than boys. Dropout rate improved (*p* < 0.05), but repetition rates increased (*p* < 0.01).
Desalegn et al. [22] (2021; Ethiopia)	Prospective cohort study	*n* = 240 SFP-beneficiary and *n* = 240 non-beneficiary children 10–14 years of age. Children were from 16 rural schools in the Sidma zone of southern Ethiopia Study duration = one academic year (initiated in 2017).	Students enrolled in the SFP schools were served a daily meal of cereals, legumes, and vegetables.	Class absenteeism Academic performance based on an aggregate score of 10 subjects.	Non-beneficiary children were two times more likely to be absent than SFP students. The average aggregate course score was 2.3 points higher among SFP students when compared to non-SFP students
Hochfeld [16] (2016; South Africa)	Pre-Post	*n* = 857 children, 6–17 years of age. Children were from six schools (five primary and one combined) in Alexandra, Johannesburg). Study duration = one year (initiated July 2011)	In-school breakfast program was initiated and implemented. Breakfast consisted of fortified cooked porridge (students in these schools already received a lunch daily).	Height, weight, BMI School performance (based off of end of term grades)	4.7% reduction in severe stunting; there was a positive change in competency scores for all grades. Improvement ranged from 3.75% for children in grade 3 to 25.79% for children in Grade R (youngest children)
Hulett et al. [23] (2014; Kenya)	CRCT	*n* = 360 students across 12 rural primary schools in the Embu district Study duration = 19 months (initiated in 1999).	Meat-githeri group 140 g of githeri and 85 g of ground beef (313 kcal) Milk-githeri group: 100 g of githeri and 250 mL of whole cows’ milk. (313 kcal) Plain-githeri group: 230 g of githeri with 3.8 g of retinol fortified oil (313 kcal)	Test scores in math, English, Kiembu, Kiswahili, geography, science, and arts.	Children in the Meat-githeri group had significantly greater improvements in tests scores than children in all other groups. Scores improved in all subjects except science. The Milk-githeri group showed greater improvements in test scores compared to the plain-githeri group and control group.
Kazianga et al. [11] (2012; Burkina Faso)	RCT	*n* = 46 newly opened schools in the Sahel region. *n* = 4236 students between 6–15 years old surveyed across schools. Study duration = one year (initiated in 2006)	SFP: lunch served on each school day (boys and girls were eligible) THR: student given 10 kg of cereal flour each month conditional on 90% attendance (girls only).	Attendance, enrollment, and cognitive development	Both the SFP and THR increased enrollment for both girls and boys. Attendance increased in students enrolled prior to the study, but it decreased in those enrolled at the start of the study. Math scores improved for girls in both programs.
Nikiema [24] (2019; Burkina Faso)	Pre-Post	*n* = 134,128 students already receiving daily meals throughout 684 schools in the Bam and Sanmatenga provinces Study duration = 9 months (initiated October 2011)	THR: 10 kg of corn-soy blend each month for girls, conditional on 90% attendance rate (schools were only eligible for the intervention if girls’ enrollment rate was under 40% and if the school was classified as rural)	Attendance, enrollment	Attendance increased by 6% for girls and by 8.4% for boys. Enrollment rates increased for girls by 3.2%. Children from schools with more female teachers benefited more from the THR intervention.
Nkhoma et al. [25] (2013; Malawi)	Pre-Post	*n* = 226 children, aged 6–8 years old, from two rural primary schools, one with an SFP and one without Study duration = one year (initiated in 2010)	SFP: children received a daily meal of corn-soy blend porridge of about 263 kcal.	Cognition (assessed via Cambridge Neurological Test Automated Battery) Weight, height, mid-upper arm circumference (MUAC)	SFP group had better scores for reversal learning, one of the brain cognitive domains. MUAC increased in the SFP group by 0.7 cm.
Omwami et al. [26] (2011; Kenya)	RCT	*n* = 554 first grade children from 12 rural schools Study duration = 2 years (84 weeks of feeding, study initiated in 1998)	Meat-githeri meal Milk-githeri meal Energy-githeri meal Meals were 240 kcal in the first year and 313 kcal in the second. All meals were served as a mid-morning recess meal	Attendance rates weight-for-age, height-for-age, and height-for-weight	Despite overall attendance decreases, intervention schools had higher attendance rates than control counterparts. Children in the Meat group had a significantly greater attendance rate than children in all other groups.

## Data Availability

No new data were created or analyzed in this study. Data sharing is not applicable to this article.

## Supplementary Materials

The following are available online at https://www.mdpi.com/article/10.3390/ijerph19063666/s1, Figure S1: PRISMA checklist.

## Appendix A

### Appendix A.1. Search Terms

The search input into each database included the terms (academic* OR education* OR learn* OR attendance* OR enrollment*) AND (“school meal*” OR “school feeding” OR “school lunch*” OR “school breakfast*” OR (school* AND (“food provisioning” OR “food services”))) AND (Africa OR Angola OR Benin OR Botswana OR “Burkina Faso” OR Burundi OR “Cabo Verde” OR Cameroon OR “Central African Republic” OR Chad OR Comoros OR Congo OR “Cote d’Ivoire” OR “Ivory Coast” OR “Equatorial Guinea” OR Eritrea OR Eswatini OR Swaziland OR Ethiopia OR Gabon OR Gambia OR Ghana OR Guinea OR “Guinea-Bissau” OR Kenya OR Lesotho OR Liberia OR Madagascar OR Malawi OR Mali OR Mauritania OR Mauritius OR Mozambique OR Namibia OR Niger OR Nigeria OR Rwanda OR “Sao Tome” OR Senegal OR Seychelles OR “Sierra Leone” OR Somalia OR “South Africa” OR Sudan OR Swaziland OR Tanzania OR Togo OR Uganda OR Zambia OR Zimbabwe).

### Appendix A.2. Risk of Bias and Quality Assessment

This review used the Academy of Nutrition and Dietetics Quality Criteria Checklist (QCC) tool to assess the risk of bias and quality of evidence [15]. Components assessed using the QCC included the research question, subject selection, group comparability, presence of withdrawals and blinding, intervention/exposure procedure, validity and reliability of results, statistical analysis, limitation and biases, and the likelihood of funding bias. Each source was evaluated for each question and given a rating of “yes” (Y) or “no” (N). If most of the answers were “no,” the article would be given a negative (-). If the answer to questions 1, 2, 3, and 4, and the majority of the remaining questions were rated “yes,” the source was rated positive (+). If the majority of questions were rated “yes,” but one of the first four received a “no”, the article was given a neutral rating (Ø). Three researchers graded each source individually, and differences were discussed until a consensus was reached. The complete evaluation of each source can be found in Table A1.

To assess the strength and quality of evidence, each source was graded based on five questions, including quality, consistency, quantity, clinical impact, and generalizability. Each source was given a rating of I-V for each question, with I being the strongest and V being the weakest. The overall grade is an average of the five subscores. The assessment procedure was the same as with the risk of bias assessment. The complete evaluation can be found in Table A2.

**Table A1:** Risk of Bias.

First Author, Year of Publication (Reference)	Alderman, 2012	Azomahou, 2019	Desalegn, 2021	Hochfeld, 2016	Hulett, 2014	Kazianga, 2012	Nikiema, 2019	Nkhoma, 2013	Omwami, 2011
**VALIDITY QUESTIONS**									
**1. Was the research question clearly stated?**	Y	Y	Y	Y	Y	Y	Y	Y	Y
**2. Was the selection of study subjects/patients free from bias?**	Y	Y	Y	Y	Y	Y	Y	Y	Y
**3. Were study groups comparable?**	Y	Y	Y	Y	Y	Y	Y	Y	Y
**4. Was method of handling withdrawals described?**	Y	Y	Y	Y	Y	Y	N	Y	Y
**5. Was blinding used to prevent introduction of bias?**	N	N	N	N	N	N	N	N	N
**6. Were intervention /exposure factor or procedure and any comparison(s) described in detail?**	Y	Y	Y	Y	Y	Y	Y	Y	Y
**7. Were outcomes clearly defined and the measurements valid and reliable?**	Y	N	Y	Y	Y	Y	Y	Y	Y
**8. Was the statistical analysis appropriate for the study design and type of outcome indicators?**	Y	Y	Y	Y	Y	Y	Y	Y	Y
**9. Were conclusions supported by results with biases and limitations taken into consideration?**	Y	Y	Y	Y	Y	Y	Y	Y	Y
**10. Is bias due to study’s funding or sponsorship unlikely?**	Y	Y	Y	Y	Y	Y	Y	Y	Y
**OVERALL QUALITY**	**+**	**+**	**+**	**+**	**+**	**+**	**−**	**+**	**+**

**Table A2:** Quality Assessment.

First Author, Year of Publication (Reference)	Alderman, 2012	Azomahou, 2019	Desalegn, 2021	Hochfeld, 2016	Hulett, 2014	Kazianga, 2012	Nikiema, 2019	Nkhoma, 2013	Omwami, 2011
**STRENGTH OF EVIDENCE ELEMENTS**									
**Quality—Scientific rigor/validity; considers design and execution**	II	III	I	II	II	I	II	II	I
**Consistency—Findings across studies**	II	I	I	II	I	II	I	III	I
**Quantity—Number of studies; number of subjects in studies**	I	I	I	I	I	I	I	II	I
**Clinical Impact—Importance of studied outcomes; magnitude of effect**	II	II	II	II	II	II	III	III	II
**Generalizability—To population of interest**	III	I	II	III	II	II	II	III	II
**OVERALL GRADE**	**II**	**II**	**I**	**II**	**II**	**II**	**II**	**III**	**I**
